# Longitudinal Associations between Internalizing Symptoms, Dispositional Mindfulness, Rumination and Impulsivity in Adolescents

**DOI:** 10.1007/s10964-021-01476-2

**Published:** 2021-07-09

**Authors:** Estíbaliz Royuela-Colomer, Liria Fernández-González, Izaskun Orue

**Affiliations:** Department of Personality, Psychological Assessment and Treatment, University of Deusto, Bilbao, Spain

**Keywords:** Dispositional mindfulness, Rumination, Impulsivity, Internalizing symptoms, Adolescents

## Abstract

Mindfulness has been associated with fewer negative mental health symptoms during adolescence, but fewer studies have examined longitudinal associations between mindfulness and symptoms in conjunction with two vulnerability factors for psychopathology with mindfulness: rumination and impulsivity. This study examined longitudinal associations between internalizing symptoms (depression, anxiety, stress), mindfulness, rumination, and impulsivity over a one-year period among 352 Spanish adolescents (57.4% girls; M = 14.47, SD = 1.34). Participants completed self-reported measures of symptoms, mindfulness, rumination, and impulsivity at two time points. Mindfulness negatively predicted stress and depressive symptoms, and a bidirectional negative association was found between mindfulness and impulsivity. Impulsivity positively predicted stress, and anxiety positively predicted depressive symptoms, stress, and rumination. This study highlights the importance of mindfulness as a protective factor and impulsivity and anxiety as risk factors for internalizing symptoms throughout adolescence. These findings build on previous studies that examined longitudinal associations between mindfulness and symptoms by including rumination and impulsivity’s roles.

## Introduction

Adolescence is a crucial developmental stage in terms of mental health difficulties (Polanczyk et al., 2015). Physiological and psychosocial changes that occur during puberty help increase internalizing symptoms—such as depression, anxiety, and stress (Graber, 2013; Romeo, 2013)—with prevalence rates higher among girls than boys (Merikangas et al., 2010). Considering that mental health problems in youth are associated with mental health problems in adulthood, both predicting and exacerbating them (Copeland et al., 2020; Johnson et al., 2018), preventing these first negative mental health symptoms during adolescence is a matter of public concern. Evidence suggests that dispositional mindfulness is associated with adolescents’ mental health and well-being (Pallozzi et al., 2017) and predicts lower levels of internalizing symptoms over time (Cortazar & Calvete, 2019). However, few studies have examined the temporal relationships between other personal factors related to mindfulness and negative mental health symptoms. This article focuses on impulsivity and rumination, considering that both constructs are associated negatively with mindfulness in adolescents (de Bruin et al., 2014; García-Rubio et al., 2019) and are viewed as transdiagnostic vulnerability factors that contribute to the etiology and maintenance of internalizing symptoms throughout adolescence (Cosi et al., 2011; McLaughlin & Nolen-Hoeksema, 2011). This study used a two-wave longitudinal design to examine the temporal bidirectional associations between mindfulness, rumination, impulsivity, and internalizing symptoms. Furthermore, the model’s sex invariance was tested.

Mindfulness is “the awareness that emerges through paying attention on purpose, in the present moment, and nonjudgmentally to the experience moment by moment” (Kabat-Zinn, 2003, p. 145). Mindfulness can be a state, training, or disposition (Brown et al., 2011). This article focuses explicitly on mindfulness as a trait or disposition that varies among individuals and can be promoted through training (Brown et al., 2011). Mindfulness theories state that acting with awareness, a core component of mindfulness, produces its salutary effects by minimizing the automatic, habitual, and impulsive reactive patterns of responding, replacing them with more conscious and adaptive responses to events (Brown et al., 2007; Williams & Kabat-Zinn, 2013). In this sense, the evidence suggests that acting with awareness is incompatible with getting stuck in repetitive thoughts, which is termed a ruminative thinking pattern (Jury & Jose, 2019). Also, it has been proposed that mindfulness reduces prepotent responses by making the individual aware of their impulsive tendencies (Peters et al., 2011). Therefore, mindfulness provides space for someone to think before they react by recognizing and having the chance to interrupt the maladaptive cognitive and behavioral patterns that increase suffering and negative mental health symptoms (Williams & Kabat-Zinn, 2013). This is particularly relevant for adolescents; thus, it seems relevant to examine the association between mindfulness and symptoms, and how they are related to other factors, namely rumination and impulsivity.

### Rumination

Rumination refers to an emotion-regulation strategy that involves responding to distress repetitively and passively focusing on its symptoms, causes, and consequences (Nolen-Hoeksema et al., 2008). It is well-documented that rumination is a cognitive vulnerability factor in adolescent psychopathology (Abela et al., 2012; McLaughlin & Nolen-Hoeksema, 2011; Wilkinson et al., 2013), particularly among girls (Rood et al., 2009). Moreover, several longitudinal studies have reported a bidirectional association between psychopathology and rumination (Jose & Weir, 2013; Krause et al., 2018). Similarly, in line with transactional psychopathology models, it has been suggested that rumination and stressors increase depressive symptoms—which, in turn, increase stress—and both predict long-term rumination (Padilla & Calvete, 2015).

As one might expect, and because a ruminative thinking pattern seems incompatible with acting with awareness, rumination is associated negatively with mindfulness (Chambers et al., 2015; Pallozzi et al., 2017; Yu et al., 2021). Indeed, a study among adolescents found that while rumination aggravated the association between stressors and internalizing symptoms, mindfulness attenuated this association (Marks et al., 2010). Furthermore, a recent study found that mindfulness reduced rumination’s impact on symptoms (Blanke et al., 2020). Together, these studies highlighted the need to address rumination as a relevant aspect associated with psychopathology and mindfulness during adolescence.

Several studies have examined rumination as an underlying mechanism through which mindfulness relates to psychological symptoms. Two cross-sectional studies among adolescents suggested that rumination mediates the association between mindfulness and symptoms. One study found that mindfulness directly impacted adolescents’ internalizing symptoms and indirectly improved their symptoms by reducing rumination (Yu et al., 2021). Similarly, another study indicated that brooding, a maladaptive form of rumination, mediated the association between mindfulness and depressive symptoms, and also was related with less awareness (Alleva et al., 2014).

Evidence from longitudinal studies is mixed. One study did not find that mindfulness protected adolescents from rumination long-term (Royuela-Colomer & Calvete, 2016), while other studies found evidence of rumination as a mediator between mindfulness and symptoms in adolescents (Ciesla et al., 2012) and adults (Jury & Jose, 2019). A similar study among adolescents found that rumination mediated the association between mindfulness and symptoms, and that this association was reciprocal (Tumminia et al., 2020). These authors emphasized the importance of studying the complex processes that arise in adolescence and comprise a synchronization of regulatory, affective, and motivational systems, considering that it is essential to understand how these cognitive-control regulatory and reactivity processes influence mindfulness. Overall, these results indicate the need to clarify longitudinal associations between negative mental health symptoms and mindfulness and rumination, including other potential factors related with symptoms, mindfulness, and rumination, such as impulsivity.

### Impulsivity

Impulsivity refers to “a predisposition toward rapid, unplanned reactions to internal or external stimuli without regard to the negative consequences of these reactions to the impulsive individuals or others” (Moeller et al., 2001, p. 1784). Impulsivity is related strongly with other concepts, such as cognitive control or executive functions, mainly inhibitory control (i.e., the capacity to inhibit an automatic or prepotent thought, emotion, or behavior in favor of a more desirable response; Diamond, 2013), and sometimes both terms are used interchangeably. Indeed, some authors have suggested that inhibitory control deficits underlie impulsivity (Horn et al., 2003; Leshem, 2016).

Much extant research supports the hypothesis that impulsivity is associated with externalizing symptoms in adolescence (Fosco et al., 2019; Stautz & Cooper, 2013). However, little research has been conducted on the association between internalizing symptoms and impulsivity. For example, a recent study reported a higher level of impulsivity in depressed adolescents compared with mentally healthy ones (Onat et al., 2019). Furthermore, several cross-sectional studies on young adults found a positive correlation between impulsivity and depressive symptoms, anxiety, and stress (Moustafa et al., 2017; Yu et al., 2020). Regarding sex differences, boys tend to be more impulsive than girls (Chapple & Johnson, 2007), and research has indicated a stronger association between impulsivity and depressive symptoms in adolescent boys than girls (Regan et al., 2019). Moreover, some researchers suggested that emotion-regulation strategies, such as rumination, mediate the association between impulsivity and depressive symptoms (d’Acremont & Van der Linden, 2007). Similarly, a longitudinal study reported an association between impulse control difficulties, mindfulness, anxiety, and depressive symptoms among college students, and this association was mediated by limited access to emotion-regulation strategies (Cheung & Ng, 2019). These studies emphasize the need to examine the association between impulsivity and symptoms during adolescence, particularly longitudinal designs.

Concerning the association between impulsivity and mindfulness, the literature suggests a negative association between these two variables (for a review Lu & Huffman, 2017). Most of these studies are cross-sectional and comprise college student samples (Lyvers et al., 2014; Maltais et al., 2020; Murphy & MacKillop, 2012; Peters et al., 2011), and to our knowledge, only one study has examined this correlation among adolescent samples (García-Rubio et al., 2019). Furthermore, three studies among adolescents reported that mindfulness was related with better inhibitory control, with both gauged using self-report and behavioral measures (Oberle et al., 2012; Riggs et al., 2015; Shin et al., 2016). Finally, some studies have suggested that impulsivity and inhibition deficits are associated with rumination in adolescence. For example, one study found that rumination was associated with difficulty inhibiting negative information (Hilt et al., 2014). Moreover, a longitudinal study reported a bidirectional association between rumination and impulsivity among college students, and that both helped intensify depressive symptoms (Hasegawa et al., 2018). Therefore, it is crucial to target impulsivity as a hallmark of psychopathology in adolescence.

So far, the studies presented highlight the need to include rumination and impulsivity when examining longitudinal associations between mindfulness and symptoms throughout adolescence, considering that both have been considered transdiagnostic factors for adolescent psychopathology (Cosi et al., 2011; McLaughlin & Nolen-Hoeksema, 2011). Furthermore, in line with transactional psychopathology models—which suggest that the associations between stress, cognitive vulnerabilities, and negative mental health symptoms are bidirectional and influence each other (Calvete et al., 2015)—it is crucial to examine how symptoms and vulnerability factors influence each other, and how they influence and are influenced by mindfulness levels. Thus, understanding longitudinal associations between mindfulness, internalizing symptoms, rumination, and impulsivity is essential to promoting mental health and developing adequate treatment strategies during adolescence.

## Current Study

This study’s main goal was to examine longitudinal associations between mindfulness, rumination, impulsivity, and internalizing symptoms (depression, anxiety, and stress) during adolescence. Based on transactional psychopathology models, a bidirectional association among all the variables was hypothesized, and the model’s sex invariance was tested. Mindfulness will predict fewer internalizing symptoms and less rumination and impulsivity (Hypothesis 1). Rumination will predict more symptoms and impulsivity, and less mindfulness (Hypothesis 2). Impulsivity will predict more symptoms and rumination, and less mindfulness (Hypothesis 3). Symptoms will predict more rumination and impulsivity, and less mindfulness (Hypothesis 4). A sex difference will exist in the predictive model: Rumination will be related strongly with the rest of the variables in girls, whereas impulsivity will be related strongly to the rest of the variables in boys impulsivity (Hypothesis 5).

## Methods

### Study Design

This study utilized a two-wave longitudinal design with a one-year time interval between Wave 1 (W1) and Wave 2 (W2). The Ethics Committee of the University of Deusto approved this study (ETK-1/19-20).

### Participants

The initial sample comprised 455 high school students between ages 11 and 18 who were enrolled in grades 7 to 11 in a private school in Vitoria-Gasteiz (Spain). From the initial sample, 352 participants (57.40% female; M_age_ = 14.47, SD_age_ = 1.34) completed the measures in the study’s two waves (permanence rate = 77.36%) and were included in the analysis. A series of *t*-tests was conducted to analyze differences in all study variables in W1 among the adolescents who completed the study’s two waves and those who failed to complete W2. Significant differences in depressive symptoms levels and age were observed. Those who failed to complete W2 were younger, [*t*(146.35) = −2.37; *p* = 0.019; *d* = 0.29] and scored higher on depressive symptoms [*t*(443) = 2.16; *p* = 0.031; *d* = 0.25]. Following Spanish Society of Epidemiology and Spanish Society of Family and Community Medicine (2000) guidelines, socioeconomic status (SES) was determined by parental occupation and education. SES distribution was as follows: 3.7% low status; 11.5% low-medium status; 35.4% medium status; 42.8% medium-high status; and 6.6% high status.

### Procedure

The school was contacted and informed about the study, and once the school principal and teachers approved it, parents were contacted to secure informed consent. The students who agreed to participate completed the questionnaires in class, including demographic data, on their computers using the Qualtrics® online platform. All data were anonymous, with unique codes assigned (i.e., comprising participants’ date of birth and parents’ first name initials) to each participant to match responses between waves. The participants completed the second assessment within a year following the same procedure.

### Materials

#### Internalizing symptoms

The Depression, Anxiety, and Stress Scale-21 Items (DASS-21; Lovibond & Lovibond, 1995) comprise 21 items distributed in three scales of seven items each: depression (e.g., “I couldn’t seem to experience any positive feeling at all”); anxiety (e.g., “I felt I was close to panic”); and stress (e.g., “I tended to overreact to situations”). Participants rated each item using a four-point scale ranging from 0 (did not apply to me at all) to 3 (applied to me very much or most of the time). The total score was computed as the mean, with scores ranging from 0 to 3. Previous studies reported good psychometric properties in young Hispanic samples (Bados et al., 2005; Román Mella et al., 2014). In this study, Cronbach’s α was 0.86, 0.81, and 0.80 in W1 and 0.88, 0.85, and 0.82 in W2 for depression, anxiety, and stress, respectively.

#### Dispositional mindfulness

The Mindful Attention Awareness Scale-Adolescents (MAAS-A; Brown et al., 2011; Calvete et al., 2014) is a 14-item self-report questionnaire that measures mindfulness as the presence of attention to and awareness of what is occurring in the present moment (e.g., “I find myself preoccupied doing things without paying attention” and “I forget a person’s name almost as soon as I’ve been told it for the first time”). Participants rated each statement using a six-point scale ranging from 1 (almost never) to 6 (almost always). The total score was computed as the mean, so scores ranged from 1 to 6. Previous studies among Spanish adolescents reported adequate psychometric properties (Calvete et al., 2014). The present study’s internal consistency was *α* = 0.80 (W1) and 0.82 (W2).

#### Rumination

The Brooding subscale from the Children’s Response Styles Scale (CRSS; Padilla & Calvete, 2011; Ziegert & Kistner, 2002) was used to evaluate rumination. The CRSS is a self-rating scale that evaluates ruminative responses to sad moods in adolescents. The Brooding subscale contains five items that assess a negative thinking pattern that involves a passive attitude toward problems and a tendency to compare an actual situation to an ideal one (e.g., “I think, ‘Why can’t I stop feeling this way?’”). Participants indicated, on a scale ranging from 1 (almost never) to 4 (almost always), the degree to which they engage in the listed thoughts when they feel sad. The total score was computed as the mean, so scores ranged from 1 to 4. The CRSS has good psychometric properties among Spanish adolescents (Padilla & Calvete, 2015). In the present study, Cronbach’s *α* was 0.72 (W1) and 0.75 (W2).

#### Impulsivity

The General Impulsivity subscale, from the Barratt Impulsiveness Scale for adolescents (BIS-11-A; Martínez-Loredo et al., 2015; Patton et al., 1995), comprises 19 items related to attentional, cognitive, and motor impulsivity. Participants reported several behaviors’ frequency levels using a four-point scale ranging from 1 (rarely/never) to 4 (almost always/always). The total score was computed as the mean, so scores ranged from 1 to 4. Sample items include “I do things without thinking” or “I am self-controlled.” Previous studies among adolescents reported strong validity and reliability (Fossati et al., 2002; Martínez-Loredo et al., 2015; Yao et al., 2007). In this study, Cronbach’s *α* was 0.74 (W1) and 0.75 (W2).

### **D**ata Analyses

Data were analyzed using IBM SPSS Statistics (IBM Corp, 2019; Version 26) and R (Version 1.3.1056; R Core Team, 2021). Each scale’s mean was computed for participants who completed at least 75% of each questionnaire’s items. Missing values were as follows: 1.4 and 6% for the DASS; 0.9 and 3.1% for the MAAS-A; 0.9 and 3.4% for the CRSS; and 0.6 and 0.9% for the Barratt, for W1 and W2, respectively. Missing values were handled through the SPSS Missing Values Analysis using the Expectation-Maximization Algorithm. Missing values were distributed completely at random and gauged using Little’s Missing Completely at Random (MCAR) test (Little, 1988): *χ*^2^ (398) = 140.73, *p* = 0.933. This indicated that MCAR may be inferred (Tabachnick & Fidell, 2013). Regarding the expected association between predictor variables, multicollinearity was examined.

Path analysis was computed using the R package Lavaan (Rosseel, 2012) and employed maximum likelihood (ML) robust standard errors and a Satorra–Bentler scaled test statistic (S–B *χ*^2^), considering that the predictor variables depressive symptoms and anxiety had a right-skewed distribution (Satorra & Bentler, 2001). The hypothesized model included cross-sectional associations between all the study variables at W1 and W2, autoregressive paths from the variables at W1 to the same variables at W2, and cross-lagged predictive paths from W1 to W2 variables. The model’s goodness of fit was evaluated using the comparative fit index (CFI), Tucker Lewis index (TLI), Akaike information criterion (AIC), root mean square error of approximation (RMSEA), and the standardized root mean square residual (SRMS). Generally, an acceptable fit is indicated by CFI, with TLI values greater than 0.95, RMSEA values below 0.06, SRMS values below 0.08, and the lowest AIC values indicating the most parsimonious model (Hu & Bentler, 1999). A multiple-group analysis was conducted to explore the invariance of the model across sex. The model first was tested separately in boys and girls, then the model’s configural invariance was tested to demonstrate that the pattern of fixed and free parameters was equivalent across subsamples. Finally, the invariance of the model’s longitudinal paths was tested.

## Results

### Descriptive Statistics and Correlation Coefficients

Table 1 displays the study variables’ descriptive statistics, including differences between boys and girls. Boys, compared with girls, scored significantly higher on mindfulness in W1 and W2, lower on anxiety and rumination in W1 and W2, and lower on depressive symptoms and stress in W2. Table 2 provides the correlations and cross-sectional covariance coefficients between all the study’s variables, which correlated significantly with each other. Mindfulness was associated negatively with internalizing symptoms, rumination, and impulsivity; rumination was related positively with impulsivity and internalizing symptoms; impulsivity was related positively with internalizing symptoms; and internalizing symptoms were related positively with W1, W2, and between waves.

**Table 1:** Descriptive statistics and sex differences for all the study variables

Variables		Total sample (*N* = 352)	Girls (*n* = 202)	Boys (*n* = 150)	Sex differences		
		M (SD)	M (SD)	M (SD)	*t*(350)	*p*	Cohen’s *d*
Depression	W1	0.83 (0.72)	0.88 (0.72)	0.77 (0.72)	1.44	0.152	
	W2	0.97 (0.76)	1.08 (0.78)	0.81 (0.69)	3.40	**0.001**	0.37
Anxiety	W1	0.83 (0.67)	0.92 (0.69)	0.72 (0.63)	2.87	**0.004**	0.30
	W2	0.92 (0.72)	1.06 (0.76)	0.72 (0.61)	4.68	**<0.001**	0.49
Stress	W1	1.20 (0.67)	1.25 (0.68)	1.14 (0.66)	1.56	0.121	
	W2	1.25 (0.67)	1.39 (0.67)	1.06 (0.62)	4.74	**<0.001**	0.51
Impulsivity	W1	1.96 (0.38)	1.95 (0.39)	1.97 (0.37)	−0.51	0.613	
	W2	1.93 (0.38)	1.92 (0.35)	1.95 (0.41)	−0.57	0.571	
Rumination	W1	2.59 (0.69)	2.71 (0.69)	2.42 (0.65)	3.95	**<0.001**	0.43
	W2	2.62 (0.70)	2.78 (0.69)	2.40 (0.64)	5.25	**<0.001**	0.57
Mindfulness	W1	4.39 (0.77)	4.31 (0.81)	4.48 (0.70)	−2.08	**0.028**	0.23
	W2	4.29 (0.77)	4.19 (0.80)	4.43 (0.69)	−2.89	**<0.001**	0.32

Significant *p* values are in bold

**Table 2:** Correlations and cross-sectional covariance coefficients between all the study variables

Variables	1	2	3	4	5	6	7	8	9	10	11	12
1. Depression (W1)	1	0.68**	0.64**	0.35**	0.51**	−0.46**						
2. Anxiety (W1)	0.65**	1	0.70**	0.31**	0.47**	−0.47**						
3. Stress (W1)	0.64**	0.68**	1	0.47**	0.48**	−0.55**						
4. Impulsivity (W1)	0.33**	0.29**	0.47**	1	0.33**	−0.57**						
5. Rumination (W1)	0.48**	0.43**	0.48**	0.33**	1	−0.50**						
6. Mindfulness (W1)	−0.45**	−0.46**	−0.55**	−0.57**	−0.50**	1						
7. Depression (W2)	0.48**	0.45**	0.35**	0.27**	0.30**	−0.37**	1	0.66**	0.55**	0.26**	0.31**	−0.38**
8. Anxiety (W2)	0.40**	0.52**	0.39**	0.20**	0.30**	−0.30**	0.73**	1	0.69**	0.23**	0.29**	−0.40**
9. Stress (W2)	0.34**	0.43**	0.48**	0.37**	0.35**	−0.44**	0.63**	0.75**	1	0.29**	0.43**	−0.47**
10. Impulsivity (W2)	0.28**	0.28**	0.35**	0.58**	0.19**	−0.42**	0.39**	0.33**	0.45**	1	0.22**	−0.42**
11. Rumination (W2)	0.32**	0.36**	0.34**	0.24**	0.46**	−0.32**	0.46**	0.43**	0.54**	0.31**	1	−0.38**
12. Mindfulness (W2)	−0.34**	−0.34**	−0.39**	−0.41**	−0.32**	0.59**	−0.47**	−0.47**	−0.61**	−0.55**	−0.47**	1

Values over the diagonal represent the cross-sectional covariance standardized coefficients obtained in the path analysis; values above the diagonal represent the Pearson correlations, except for Depression and Anxiety variables because they were not normally distributed and Spearman correlations are presented

**p < 0.01

### Predictive Model

**Fig. 1:**
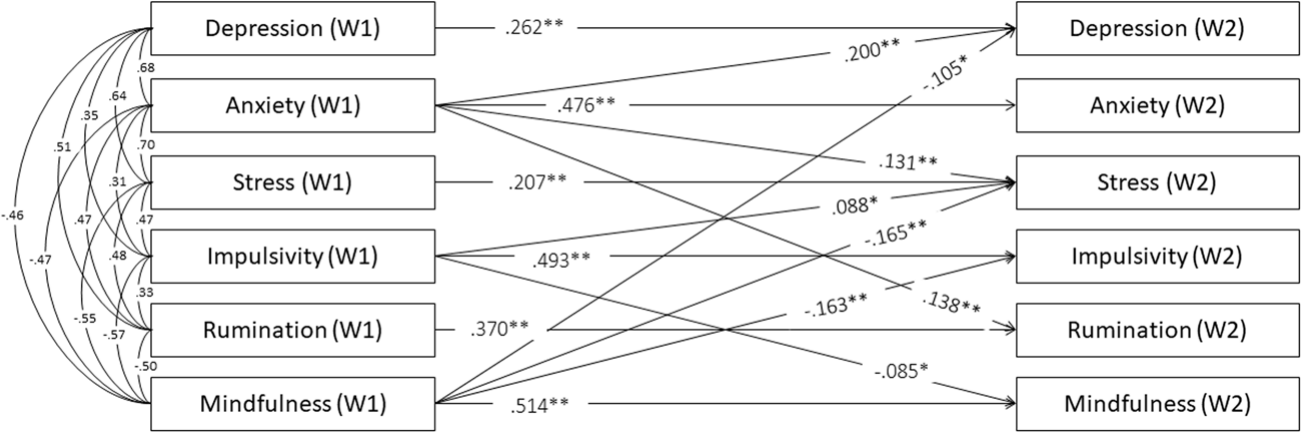
Statistically significant longitudinal paths between symptoms, dispositional mindfulness, rumination and impulsivity and cross-sectional covariance standardized coefficients. Longitudinal paths: *p < 0.05. **p < 0.01. All cross-sectional associations were significant *p* < 0.001

No sign of multicollinearity was detected, with all tolerances above 0.40 and variance inflation factors (VIF) <2.5. At the longitudinal level, all the autoregressive paths were statistically significant, indicating these variables’ stability over the one-year follow-up, as 0.26, 0.48, 0.21, 0.49, 0.37, and 0.51 for depressive symptoms, anxiety, stress, impulsivity, rumination, and mindfulness, respectively. Figure 1 provides the model’s cross-lagged regressive coefficients that were statistically significant. Mindfulness predicted less depressive symptoms (*β* = −0.11, *p* = 0.030), stress (*β* = −0.17, *p* < 0.001), and impulsivity (*β* = −0.16, *p* = 0.002), but mindfulness did not predict anxiety or rumination significantly. Impulsivity negatively predicted mindfulness (*β* = −0.09, *p* = 0.045) and positively predicted stress (*β* = 0.09, *p* = 0.033), but impulsivity was not associated with depressive symptoms, anxiety, or rumination. Regarding internalizing symptoms, depressive symptoms and stress did not longitudinally predict any variable of the model. Nevertheless, anxiety predicted higher levels of depressive symptoms (*β* = 0.20 *p* = 0.001), stress (*β* = 0.13, *p* = 0.008), and rumination (*β* = 0.14, *p* = 0.010), but it did not predict impulsivity or mindfulness in W2. A more parsimonious model was estimated, excluding insignificant paths, with adequate fit indices: S–B *χ*^2^ (21, *N* = 352) = 25.074; *p* = 0.244; RMSEA = 0.024; 90% CI [0.000, 0.053]; CFI = 0.998; TLI = 0.994; SRMR = 0.052; and AIC = 5942.930. The model explained 24, 23, 23, 33, 20, and 32% of the variance in depressive symptoms, anxiety, stress, impulsivity, rumination, and mindfulness in W2, respectively.

### Differences between Girls and Boys for the Predictive Model

Using multiple-group analysis, it was examined whether the model was invariant in girls and boys. First, the model was estimated separately in girls—S–B *χ*^2^(11, *N* = 202) = 16.517, *p* = 0.123, RMSEA = 0.049, 90% CI [0.000, 0.094], CFI = 0.996, TLI = 0.972, SRMR = 0.027, and AIC = 6103.675—and boys—S–B *χ*^2^(11, *N* = 150) = 10.435, *p* = 0.492, RMSEA = 0.000, 90% CI [0.000, 0.083], CFI = 1, TLI = 1.004, SRMR = 0.022, and AIC = 4319.852. Second, the configural invariance of the pattern of fixed and free model parameters across sex subsamples was tested (this model included the significant paths obtained for boys, girls, and the complete sample): S–B *χ*^2^(38, *N* = 352) = 45.985; *p* = 0.175; RMSEA = 0.035; 90% CI [0.000, 0.067]; CFI = 0.996; TLI = 0.988; SRMR = 0.041; and AIC = 5970.061. Finally, the invariance across sex of the model’s predictive paths was tested, and it did not increase S–B *χ*^2^ significantly [∆S–B *χ*^2^ (17, *N* = 352) = 19.917, *p* = 0.279], thereby indicating that the overall path pattern was invariant between girls and boys.

Although these results indicate that the model was invariant across sex, it was tested whether a significant difference in the model’s paths existed between girls and boys for exploratory purposes. The lavTestScore function in the Lavaan R package (Rosseel, 2012) was used, providing expected parameter changes (EPCs) if model constraints are released. This test’s results indicated a difference in the autoregressive path of mindfulness [∆S–B *χ*^2^(1, *N* = 352) = 4.8511, *p* = 0.028] and at the cross-lagged regression path between impulsivity in W1 and depressive symptoms in W2 [∆S–B *χ*^2^ (17, *N* = 352) = 4.2169, *p* = 0.040]. Specifically, the association between mindfulness in W1 and W2 was stronger in girls compared with boys (Beta = 0.54, *p* < 0.001 and Beta = 0.39, *p* < 0.001 in girls and boys, respectively), and the association between impulsivity in W1 and depressive symptoms in W2 was non-significant for girls, but significant for boys (Beta = −0.00, *p* = 0.998 and Beta = 0.30, *p* = 0.008 for girls and boys, respectively).

### Sensitivity Analysis

The primary analyses were replicated with a log transformation for depressive symptoms and anxiety predictor variables, as they presented a right-skewed distribution. This transformation did not alter the results, which are presented in the Supplementary Material.

## Discussion

Previous studies suggest that mindfulness predicts fewer internalizing symptoms in adolescents (Cortazar & Calvete, 2019). According to mindfulness theories, acting with awareness reduces automatic, habitual, impulsive cognitive and behavioral responses (such as rumination and impulsivity) by facilitating a more flexible and adaptive response to events (Brown et al., 2007; Williams & Kabat-Zinn, 2013). Nevertheless, when studying longitudinal associations between mindfulness and negative mental health symptoms, few studies have included its associations with rumination and impulsivity, which are viewed as transdiagnostic factors for psychopathology throughout adolescence (Cosi et al., 2011; McLaughlin & Nolen-Hoeksema, 2011). Therefore, this study examined, over a one-year period, longitudinal associations between mindfulness, rumination, impulsivity, and internalizing symptoms (depressive symptoms, anxiety, and stress) in adolescents and whether the model was sex invariant.

Several interesting findings emerged from the autoregressive, cross-lagged path analysis. First, mindfulness prospectively predicted less stress, depressive symptoms, and impulsivity, but not anxiety or rumination. Second, rumination did not predict any variable in W2. Third, a bidirectional negative association between mindfulness and impulsivity was found. Fourth, impulsivity positively predicted stress, but not rumination, anxiety, or depressive symptoms. Concerning internalizing symptoms, only anxiety predicted other symptoms (i.e., depressive symptoms and stress) and rumination in W2. These results were observed controlling for all variables in W1, i.e., the associations between variables in W1 and W2 are not spurious due to their associations in W1. Thus, the model was sex invariant.

In line with previous studies, this study found that mindfulness was negatively correlated cross-sectionally with internalizing symptoms, rumination, and impulsivity (García-Rubio et al., 2019; Pallozzi et al., 2017; Tan & Martin, 2012). Regarding longitudinal associations, the results partially support the hypothesis. Consistent with previous longitudinal studies among youth, mindfulness predicted fewer depressive and stress symptoms and impulsivity in the one-year follow-up (Cheung & Ng, 2019; Cortazar & Calvete, 2019; Royuela-Colomer & Calvete, 2016). It seems that acting with awareness could make adolescents less impulsive and less susceptible to depressive and stress symptoms. Furthermore, individuals high in mindfulness could be more able to notice how the mind reacts to thoughts, sensations, and information, recognizing and avoiding habitual patterns that unconsciously guide behavior, thereby selecting better and healthier ways of responding to experiences. Indeed, some studies suggested that mindfulness buffers stressors’ impact on depressive symptoms among adolescents (Thomas et al., 2021). Similarly, considering that previous studies reported that mindfulness predicts a reduction in stressors in the long term among adolescents (Calvete et al., 2017), it could be that having fewer stressors reduces stress and depressive symptoms.

However, contrary to expectations, mindfulness did not predict anxiety or rumination in the long term. Although a previous study among adults reported a longitudinal association between mindfulness and rumination (Jury & Jose, 2019), a study among adolescents did not find a longitudinal association between the acting-with-awareness facet of mindfulness and rumination (Royuela-Colomer & Calvete, 2016). Thus, this discrepancy might suggest a developmental difference in rumination’s role. Furthermore, the results did not indicate that mindfulness predicts anxiety, which contradicts some previous findings (Cortazar & Calvete, 2019). One reason could be that these authors included a combined measure of depressive symptoms and anxiety, and did not examine the effect from mindfulness for each symptom individually. Thus, it is possible that when a longitudinal association between mindfulness and internalizing symptoms is analyzed for each symptom separately, it is significant for some symptoms (stress and depressive symptoms), but not for others (anxiety). Furthermore, this also could suggest an interaction between mindfulness and stress, e.g., individuals with low mindfulness levels and high stress levels could be at higher risk of experiencing more anxiety symptoms. In support of this idea and similar to the results, a recent study did not find a direct longitudinal association between mindfulness and anxiety (Cheung & Ng, 2019). This finding could suggest that other mechanisms or facets of mindfulness, such as non-judging inner experience or non-reactivity, could influence the longitudinal association between mindfulness and anxiety symptoms.

Although rumination was associated cross-sectionally with all the variables, the path analysis results indicate that rumination was not associated longitudinally with any variable. Despite rumination’s importance as a cognitive vulnerability factor for psychopathology in adolescence (McLaughlin & Nolen-Hoeksema, 2011), one study found that rumination longitudinally did not predict internalizing symptoms over and above previous symptoms and mindfulness (Schut & Boelen, 2017). Indeed, this could explain why rumination did not predict the rest of the variables, with the results indicating that rumination in W1 does not explain W2 variables’ variability over and above the rest of the model’s variables. Thus, rumination’s maladaptive role may depend on interaction with other variables. For instance, some studies proposed that rumination could act as a moderator, worsening negative mental health symptoms, e.g., when combined with stressors or previous symptoms (Abela & Hankin, 2011; Ciesla et al., 2012; Cohen et al., 2014; Marks et al., 2010).

For impulsivity, as previous studies suggested (Cheung & Ng, 2019; Maltais et al., 2020; Peters et al., 2011), the results confirm a cross-sectional and longitudinal association between impulsivity and mindfulness. Interestingly, this study builds on previous literature using cross-sectional designs by indicating that this association is bidirectional. The obtained results suggest that mindfulness might reduce impulsivity in the long term, and simultaneously, higher impulsivity levels might decrease mindfulness in the long term. Surprisingly, contradicting the hypothesis, impulsivity did not predict depressive symptoms, possibly because other underlying mechanisms influence the association between impulsivity and depressive symptoms. For instance, the results could suggest an indirect effect from impulsivity on depressive symptoms by reducing mindfulness, i.e., impulsivity might predict more depressive symptoms in the long term by lowering mindfulness levels. It should be noted that although the model was invariant across sex, impulsivity predicted depressive symptoms only in boys. Another study also reported a stronger association between impulsivity and depressive symptoms in boys (Regan et al., 2019). As these authors proposed, boys and girls might experience depressive symptoms differently, and the present study’s results suggest that impulsivity predicts more depressive symptoms in boys. Thus, the sex differences highlight the importance of targeting impulsivity as a risk factor in boys’ depressive symptoms.

Interestingly, it was found that impulsivity predicted more stress symptoms in the long term. Considering that impulsivity is associated commonly with maladaptive behaviors in adolescence—such as alcohol abuse, delinquency, and risky behaviors (Fosco et al., 2019; Romer, 2010; Stautz & Cooper, 2013)—it could be that more impulsive adolescents engage in maladaptive behaviors, thereby increasing stressors and stress symptoms. To our knowledge, this is the first longitudinal study that has examined the association between impulsivity and stress symptoms among adolescents, but future longitudinal studies are needed to understand these results and examine whether other mechanisms are influencing this association.

While depressive symptoms and stress did not predict other variables longitudinally, anxiety symptoms predicted depressive and stress symptoms and rumination. Thus, it can be suggested that anxiety symptoms are a risk factor for other internalizing symptoms and rumination in the long term. The explanation of anxiety as a risk factor is consistent with direct causal anxiety models, suggesting that anxiety precedes depression in adolescence (Mathew et al., 2011), and the results extend this to stress and rumination. Indeed, a limitation from previous studies is that internalizing symptoms are studied as a unitary construct, or only including depressive symptoms, and that depression and anxiety are different and follow different developmental trends (Graber, 2013). Specifically, it has been suggested that anxiety increases throughout childhood, while depressive symptoms increase during adolescence (Graber, 2013). The findings also suggest that anxiety predicts rumination, with previous studies indicating that rumination could be viewed as a transdiagnostic risk factor during adolescence (McLaughlin & Nolen-Hoeksema, 2011). However, rumination did not predict any variable in W2, indicating that rumination’s maladaptive role could be better explained with a diathesis-anxiety model (Cohen et al., 2014). Such models suggest that anxiety interacts with cognitive vulnerabilities, such as rumination, to predict other symptoms. Therefore, it could be that adolescents with high anxiety levels, when combined with rumination, are at a higher risk of depressive symptoms. Future studies should test this hypothesis. This study’s findings are interesting for extending knowledge on developmental models of psychopathology and support previous evidence that anxiety must be a target risk factor that predicts psychopathology.

Contrary to expectations, this study did not find a significant sex difference in the predictive model, but sex differences were found in the variables. As suggested in the literature (Brown et al., 2011; Graber, 2013; Rood et al., 2009), the results indicate that girls have higher anxiety and rumination levels, and lower mindfulness levels at both time points, whereas differences in depressive symptoms and stress are only present in W2. One reason that might explain the absence of sex differences for depressive symptoms and stress in W1 could be because, as opposed to sex differences in rumination and anxiety appearing earlier during development (Graber, 2013), sex differences in depressive symptoms appear later, during adolescence, and it could be the same for stress symptoms. It also should be noted that, as opposed to previous studies (Chapple & Johnson, 2007), no sex differences in impulsivity were found at any time point. However, the results indicate that the predictive association between impulsivity and depressive symptoms was significant only in boys, suggesting that impulsivity could be a mechanism underlying depressive symptoms in boys, but not in girls.

The most important limitation in this study is attrition rate. Regarding the longitudinal nature of the research and the long time interval studied (1 year), some participants failed to complete the second assessment. Moreover, significant differences in depressive symptom levels and age were found between those who completed both time waves and those who only completed W1, compelling us to be cautious about generalizing these results to younger adolescents with higher depressive symptoms levels. A second limitation is that all participants were from only one high school, and most had medium/high SES, limiting the results’ generalizability. Third, this study used only two time points, and future studies should use at least three time points to examine mediation mechanisms. Finally, it should be noted that, similar to other studies’ results, *r*-square coefficients were low (Cortazar & Calvete, 2019; Schut & Boelen, 2017). Because adolescence is a complex developmental period, other contextual factors, such as maturation processes or stressors, also could have impacted outcome variables, and future studies should consider other variables. Despite these limitations, the current study has several strengths. First, this study’s longitudinal nature enables examination of bidirectional associations over a one-year period, building on results from previous cross-sectional studies. Second, this research was based on a sample of high school adolescents and builds on previous studies’ knowledge using college student samples. Third, this study builds on previous longitudinal studies that assessed the relationships between mindfulness and negative mental health symptoms by including the associations with two important factors for adolescent development and mindfulness theories: rumination and impulsivity.

## Conclusion

Impulsivity and rumination are transdiagnostic vulnerability factors that contribute to the etiology and maintenance of negative mental health symptoms throughout adolescence, and both constructs are associated with mindfulness. However, the longitudinal associations between mindfulness, rumination, impulsivity, and internalizing symptoms during adolescence are not clear. Over the course of 1 year, the current study examined the relationships between internalizing symptoms (depression, anxiety and stress), mindfulness, rumination and impulsivity in youth. The results suggest that mindfulness protects against stress and depressive symptoms, but not against anxiety, when controlling for the rest of the model’s variables. In addition, this study’s findings indicate that anxiety symptoms could be a risk factor for depressive and stress symptoms and rumination. Specifically, adolescents with higher anxiety levels could ruminate more in the long term. Interestingly, anxiety was not predicted by any variable in the model, indicating that anxiety might precede other symptoms during adolescence. Finally, this study found a bidirectional association between impulsivity and mindfulness, indicating that both influence each other in the long term. Moreover, the results suggest that those with higher impulsivity levels might be at greater risk of developing stress symptoms. The results correspond with those from previous studies, evidencing the protective role of mindfulness during adolescence, specifically for depression and stress symptoms. Overall, the results underscore the importance of carrying out intervention programs that promote mindfulness in youth. Similarly, it is important to examine whether these interventions protect against stress and depressive symptoms by reducing impulsivity.

## Supplementary Information

Supplementary Material
