# Supplementary material for: Longitudinal Associations between Internalizing Symptoms, Dispositional Mindfulness, Rumination and Impulsivity in Adolescents
Source: J Youth Adolesc. 2021 Jul 9;50(10):2067–78. doi: 10.1007/s10964-021-01476-2 (PMC8416885; doi:10.1007/s10964-021-01476-2)
Supplement: Supplementary file 1 — Supplementary Material [file 10964_2021_1476_MOESM1_ESM.docx]

**Supplementary Material**

**Sensitivity Analysis**

A logarithmic transformation was applied to depression and anxiety predictor variables, since both presented a right skewed distribution. The x'=log(x+1) transformation was used because both variables included zero values. A complete model was estimated that included all autoregressive paths, cross-sectional associations and cross-lagged paths. Then a more parsimonious model that excluded non-significant paths was estimated. This model had adequate fit indexes: S-B χ2(21, N = 352) = 29.347, *p* = .106, RMSEA = .034, 90 % CI [.000, .060], CFI = .996, TLI = .989, SRMR = .054, AIC = 5015.353. Results from the path analysis indicated that at the longitudinal level, all the autoregressive paths were statistically significant, indicating these variables' stability over the one-year follow-up, and were: .27, .48, .19, .49, .37, .52 for depression, anxiety, stress, impulsivity, rumination, and mindfulness, respectively. Mindfulness predicted less depression (β = -.10, *p* = .030), stress (β = -.16, *p* < .001) and impulsivity (β = -.16, *p* = .002), but mindfulness did not significantly predict anxiety or rumination. Impulsivity negatively predicted mindfulness (β = -.08, *p* =.048) and positively stress (β = .09, *p* = .033); however, impulsivity was not associated with depression, anxiety, or rumination. Regarding internalizing symptoms, depression and stress did not longitudinally predict any variable of the model. Nevertheless, anxiety predicted higher levels of depression (β = .20 *p* = .001), stress (β = .16, *p* = .002), and rumination (β = .14, *p* = .008), but it did not predict impulsivity or mindfulness at W2. The model explained 24, 23, 24, 33, 20, 32% of the variance, of depression, anxiety, stress, impulsivity, rumination, and mindfulness at W2, respectively.
